# *Aloe ferox* as a Candidate Botanical Insecticide for Stored-Grain Protection: Evidence, Knowledge Gaps, and Prospects for *Sitophilus zeamais* Management

**DOI:** 10.3390/molecules31183190

**Published:** 2026-09-10

**Authors:** Florence Bukky Aina, Lisa Buwa-Komoreng, Lelethu Unathi-Nkosi Peter Heshula, Nyasha Esnath Chiuta, Tolulope Olubunmi Adeniji, Siphamandla Lamula, Shadreck Muchaku, Mhlangabezi Slayi, Charles Shelton Mutengwa

**Affiliations:** 1 Department of Agronomy, University of Fort Hare, Private Bag X1314, Alice 5700, South Africa; nyashachiuta@gmail.com (N.E.C.); cmutengwa@ufh.ac.za (C.S.M.); 2 SAMRC Microbial Water Quality Monitoring Centre, University of Fort Hare, Private Bag X1314, Alice 5700, South Africa; lheshula@ufh.ac.za (L.U.-N.P.H.); tolbum17@gmail.com (T.O.A.); 3 DSTI/NRF SARChI Chair in Water Quality and Environmental Genomics, University of Fort Hare, Private Bag X1314, Alice 5700, South Africa; 4 Department of Botany, University of Fort Hare, Private Bag X1314, Alice 5700, South Africa; lbuwa@ufh.ac.za; 5 Infectious Diseases and Medicinal Plants RNA, University of Fort Hare, Private Bag X1314, Alice 5700, South Africa; slamula@ufh.ac.za; 6 Department of Zoology & Entomology, University of Fort Hare, Private Bag X1314, Alice 5700, South Africa; 7 Centre for Global Change, University of Fort Hare, Private Bag X1314, Alice 5700, South Africa; muchakushadreck@gmail.com (S.M.); mslayi@ufh.ac.za (M.S.)

**Keywords:** biopesticides, phytochemicals, secondary metabolites, postharvest protection, anthraquinones, botanical pest management, integrated pest management

## Abstract

Botanical insecticides are increasingly being investigated as alternatives to synthetic pesticides for stored-grain protection. This review critically evaluates *Aloe ferox* Mill. as a candidate botanical insecticide with potential relevance to *Sitophilus zeamais* Motsch, the major stored-maize pest. The available evidence related to the phytochemistry, reported pesticidal activities, proposed mechanisms of action, safety, and research needs was synthesised. *A. ferox* possesses a variety of secondary metabolites like anthraquinones, flavonoids, phenolics, chromones, tannins, alkaloids, and saponins, some of which have been reported to possess insecticidal, repellent, antifeedant, or growth-regulatory activity in other biological systems. But there is currently no direct experimental proof of the effectiveness of *A. ferox* against *S. zeamais*. A large proportion of the evidence relevant to pesticides is from other *Aloe* species, other insect and arthropod pests, or from one component or phytochemical studied in a different biological system. The findings, therefore, offer a justification for further investigations, but do not provide evidence of efficacy. Future research should focus on bioassays that directly test dose–responses, phytochemical standardisation, mechanism validation, formulation development, grain quality and residue evaluation, safety evaluation, and validation using realistic storage conditions.

## 1. Introduction

Botanical insecticides, which are plant-derived, may be an appropriate alternative to synthetic insecticides because they are environmentally friendly, generally species-specific, inexpensive, biodegradable, less vulnerable to insect resistance, and non-toxic to humans [1,2]. They also have various modes of action, providing quick knockdown and helping reduce storage losses. The following plants have been used as sources for commercialising botanical pesticides: *Schoenocaulon officinale*, *Azadirachta indica*, *Tanacetum cinerariifolium*, *Ryania speciosa*, and *Nicotiana tabacum* [1,3,4].

The misuse and excessive use of pesticides have led to a variety of environmental and health issues, as well as impacts on non-target species [5]. Only 1–25% of the pesticide applied reaches the target pests, while the rest enters neighbouring ecosystems, leading to soil degradation, water pollution, biodiversity loss, and greenhouse gas emissions [2]. According to the World Health Organisation, 200,000 people around the world are killed each year as a direct result of the effects of pesticides [4]. The risk of human fatality from the use of pesticides has been reported as being highest in Africa [4]. Ample evidence shows a carcinogenic risk associated with pesticide use. Major chronic health effects of pesticides include neurological, respiratory, and reproductive effects; cardiovascular problems; gastrointestinal disturbances; and cancer [6,7]. These health impacts are chronic and may not be detected until several years after exposure, highlighting the need for strict regulations and improved management practices [6]. Although many reviews have been published on botanical insecticides, no comprehensive review specifically focuses on the potential of *A. ferox* as a botanical insecticide for controlling *S. zeamais*. This review synthesises existing information on its phytochemistry, reported pesticidal activities, possible mode of action, safety concerns, and future research needs.

*S. zeamais* Motsch. (1855) (Coleoptera: Curculionidae) stands out as the main pest of stored grains, responsible for significant deterioration of stored products, and is particularly important in maize crops [8,9,10]. The maize weevil infests a wide range of cereals and their products, resulting in weight reduction, depreciation of commercial value, reduction in nutritional value, and secondary causes such as the emergence and dissemination of fungi due to the increase in moisture of the grain mass as a result of the high density of insects [11]. *S. zeamais* cause qualitative and quantitative damage to stored products, ranging from 20 to 90% reduction in grain weight resulting from untreated stored maize [9,12,13].

Postharvest losses refer to the loss of edible food in the food value chain, including production, storage, transportation, processing, and marketing, before it reaches the market [14,15]. Postharvest losses are among the biggest problems for food security in the developing world and can occur at any point in the food chain, from harvest to consumption [16]. Reducing postharvest losses is one practical way to boost agricultural output and sustainably stabilise the food supply [17]. Postharvest loss exacerbates the nation’s food insecurity problem by reducing the amount of food available, which raises food prices [18].

Rather than completely replacing synthetic pesticides, plant insecticides are increasingly regarded as complementary components in integrated pest management systems. Taken together, recent studies suggest that plant-based pesticides offer several benefits, such as biodegradability, lower environmental persistence, multiple modes of action, and generally lower resistance potential. However, these benefits are not universal, as the effectiveness of botanical insecticides can vary greatly among plant species, phytochemicals, extraction processes, formulations, and target pests. In addition, synthetic pesticides are highly effective for rapid pest control but raise significant concerns about environmental contamination, non-target toxicity, and the development of resistant insect populations. These issues have sparked a search for plant compounds with strong pest-control and environmental-safety properties. Therefore, screening plants with diverse secondary metabolites and bioactive properties, such as *A. ferox*, has become increasingly important for finding sustainable alternatives for stored-grain protection and meeting food security requirements. The aim of this review is to critically address the phytochemical composition, as well as reported insecticidal activity, mechanisms of action, safety profile, and gaps in the knowledge of *A. ferox* for use as a botanical insecticide in the sustainable management of *S. zeamais* in stored grains.

## 2. Literature Search Methodology

This study aimed to explore the potential of *A. ferox* as a botanical insecticide for stored-grain pests, especially *S. zeamais*, through a critical literature review. The literature reviewed was found mostly using Google Scholar and Scopus. The search was non-systematic and based on keyword combinations aligned with the review’s goals and major topics. The keywords used were “*Aloe ferox*”, “*Aloe vera*”, “*Aloe* species”, “botanical insecticides”, “biopesticides”, “plant-derived pesticides”, “phytochemicals”, “secondary metabolites”, “*Sitophilus zeamais*”, “maize weevil”, “stored-grain protection”, “postharvest pest management”, “repellent activity”, “antifeedant activity”, “larvicidal activity”, “acaricidal activity”, “integrated pest management”. We used the Boolean operators AND and OR as needed to link keywords. The search was conducted as a narrative and exploratory review, so no single search string was used across databases.

No year limit was placed on the literature; both recent and past literature were reviewed. Earlier publications were included as they contained background information on the taxonomy, traditional use, phytochemical composition, biological activities, or insecticidal properties of the *Aloe* species, while more recent publications were included if they were particularly relevant to the current developments of botanical insecticides, stored-grain protection, safety, and formulation. During the literature search, no intention was made to limit the language; however, the literature that was considered in the review was mainly written in English and accessible to the authors.

The reviewed literature was selected mainly from peer-reviewed journal articles that contained information about the phytochemicals, biological activity, insecticidal activity, action mechanisms, toxicity and safety of *A. ferox* and related *Aloe* species, as well as the environmental impact. Other species of Aloe were included in the studies where there was no direct evidence of the *A. ferox* effect, and were employed as indirect evidence of the insecticidal activity of *A. ferox*. Likewise, research with stored-product insects was given higher priority; other insects or arthropods of economic significance were included only when they supplied data relevant to supporting information on the biological activity or potential mode of action of compounds produced by *Aloe*. In the synthesis, this distinction was retained to prevent the blending of evidence from other *Aloe* species or unrelated arthropods with that of *S. zeamais* and *A. ferox*.

The reference lists of key articles that were retrieved were examined to locate additional relevant publications. Relevant studies were identified through backward citation searching, which may not have been identified in the original keyword searching. Relevant publications that were scientifically sound and useful for understanding the phytochemistry, biological activity, insecticidal activity, safety, and application potential of *A. ferox* and related aloe species were chosen. Peer-reviewed journal articles were given a higher level of priority, although seminal articles were included where they were crucial for foundational and/or contextual information.

This was a narrative review, and therefore there was no application of a formal systematic review protocol, no application of a predefined systematic review framework (PRISMA), and no quantitative risk of bias assessment and quality scoring system. Therefore, no prospective records were recorded for record-by-record screening, the number of records screened, or the reasons for excluding individual records. The review is not intended to be a systematic or comprehensive review of the literature. Instead, the evidence identified was critically and thematically synthesised to assess existing knowledge, gaps, limitations, and future research prospects for developing *A. ferox* as a botanical insecticide for stored-grain protection.

## 3. Biology and Economic Importance of *S. zeamais*

*S. zeamais* is an important storage pest of maize and is a cosmopolitan stored-product pest that can also start an infestation in the field. It affects nutritional quality and impacts the commercial and agronomic value and sensory properties of stored grains such as maize, wheat, rice, and sorghum [19,20,21]. Maize infestation can reduce the nutritional value, germination rate, and weight. A single *S. zeamais* weevil can cause a 35 mg weight loss in a maize kernel from development to the adult stage [19]. Maize weevils significantly hamper food security through postharvest losses. Maize weevils can cause 90% losses in extreme infestations [22]. Stored grain quality depends on many factors, including inadequate light, humidity, temperature, and time since harvest, as well as biotic factors such as fungi, bacteria, rodents, and insects [23].

Early detection is difficult in *S. zeamais* because females lay eggs in grain-boring holes, and pest management is complicated by multiple generations within a single season. Adults and larvae cause damage to grain. Adults bore through the grain to deposit eggs, which hatch into larvae after 5–6 days. The larvae feed on the endosperm of the grain and enter the pupal stage. Larvae are white, stout, and dense, with no legs, and they possess a brown head that is approximately 4 mm long. The larval stage lasts approximately 25–30 days, and the pupal stage lasts 5–8 days (average 6). Once pupae turn into adults, they bore into the grain and emerge into the environment. Pupae are white and 3–4 mm long. Adults live 4–5 months when food is available and about 36 days without food. During a female’s reproductive life, she can lay as many as 250 eggs, with a maximum of 300 eggs. The life cycle is around 30–45 days at 29 °C, 70% RH, and a seed moisture content of 14%. If the moisture content of the material in storage exceeds 15%, then the population increases very rapidly. In temperate zones, there are two to three generations per year [24,25,26].

The biological features of *S. zeamais* are, to a great extent, responsible for its status as one of the most destructive pests of stored cereals in the world. The immature stages develop within the grain kernel and are not exposed to environmental stresses or direct contact with insecticides, unlike those of many other external feeders. Populations build up rapidly before infestations become apparent because of hidden development, high reproduction rates, overlapping generations, and a relatively long adult lifespan under good storage conditions. Furthermore, the relationship between insect infestation and inadequate storage conditions forms a vicious cycle in which insect feeding leads to greater grain damage and moisture, which, in turn, allows more fungal contamination and further damage. Together, these measures indicate that control must target several developmental stages of the insect life cycle, not just the adult stage of *S. zeamais*.

## 4. Botanical Insecticides in Stored-Grain Protection

The dried flowers of *Tanacetum cinerariifolium* (Asteraceae) are the source of pyrethrum, the most widely used botanical insecticide worldwide, although the precise amount used is difficult to determine. In recent years, pyrethrin use for crop protection has increased [27]. The following plant species: *Maesa lanceolata*, *Croton macrostachyus*, *Carica papaya*, *Calpurnia aurea*, *Clausena anisata*, *Vernonia amygdalina*, *Chenopodium* sp., and *Nicotiana* sp. have been used to control the maize weevil [16,28]. In the case of pulse beetles, it was observed that all of the plants’ leaf extracts (*Prosopis* spp., *Nerium* spp., *Ocimum* spp., *Acalypha* spp., *Catharanthus* spp., and *Vitex* spp.) were found to have a noticeable ovipositional deterrent effect [29]. Several plants, including *Ryania speciosa*, *Schoenocaulon officinale*, *Nicotiana tabacum*, *Tanacetum cinerariifolium*, and *Azadirachta indica*, have already been exploited and commercialised as botanical pesticides [1].

Essential oils (EOs) are gaining popularity as a promising biorational strategy to control stored insect pests of maize, particularly *S. zeamais*, as fumigants and contact insecticides, with demonstrated efficacy [30]. The buds, flower petals, stem rhytidome, leaves, seeds, roots, resins, and fruit peels of the plant are sources of EOs [30]. Previous investigations demonstrated the potential of EOs to control *S. zeamais* through toxic (contact, ingestion, and fumigation) and behavioural (feeding deterrence, repellency, and inhibition of oviposition and growth) effects [31,32].

Secondary metabolites in plants are crucial for their natural defence against pests, diseases, and abiotic stressors [33]. As natural products, they are biodegradable, less harmful to people, and participate in a variety of activities. Depending on their chemical structure, plant secondary metabolites fall into four main groups: phenolics, nitrogen-containing compounds, terpenes, and sulphur-containing compounds [33]. Although plant products have been extensively tested in laboratories worldwide, only a few have passed the registration process for use in the storage sector. Botanical extracts are complex chemical mixtures that may disrupt the nervous systems of stored-product pests, as well as their reproduction or behaviour [34].

The body of evidence shows that botanical insecticides act through multiple, complementary modes of action rather than a single toxic mechanism. Botanical products can cause mortality via contact toxicity and fumigation, and can also affect development, reproduction, oviposition, and feeding, depending on the plant species and their phytochemical composition. Multiple bioactive constituents may provide several modes of action, although the mechanism depends on the botanical preparation and target pest. However, published studies are difficult to compare directly because experimental conditions, extraction processes, application rates, storage conditions, and target insect species vary widely. Hence, despite the consistently good insecticidal activity demonstrated in laboratory studies, there is still a long way to go towards reliable field or commercial use in storage systems, necessitating improvements in the standardisation of extraction protocols, efficacy testing, and product formulation.

## 5. Botanical Description and Economic Importance of *A. ferox* and Related *Aloe* Species

The *Aloe* species belong to the family Asphodelaceae [35]. *Aloes* are perennial plants, succulent forbs, shrubs, and trees that may grow up to 3300 m above sea level in a range of environments [36], and most of them may be identified morphologically by their rosettes of thick, fleshy, frequently sword-shaped, spiky leaves that end at the trunk or branches [37]. The blooms, which are primarily tubular but occasionally bell-shaped in some species, are typically vividly coloured and grouped in simple or branched inflorescences resembling candles or cones. Most fruits are dry, dehiscent capsules that release winged seeds [36].

They have been utilised as a laxative, antiulcer, immunostimulant, purgative, antiseptic, emmenagogue, and remedy for inflammatory conditions, wound and burn healing, antitumor activity, gastrointestinal and skin issues, microbial infections, and antidiabetic activity [36,38]. They have antibacterial and cell-proliferative properties; hence, they have been utilised to treat wounds and burns [36]. Aloe leaves contain two components: the gel and the leaf exudate. *Aloe* leaf gel is often used in traditional medicine as a skin treatment for wounds and sunburns. The polysaccharides and glycoproteins of this gel are believed to contribute to wound-healing activity and are present in the inner leaf. The bitter leaf exudate has been recognised for its laxative effect [35]. *Aloe* has been used medicinally to treat various diseases, as a food component and in cosmetics for hundreds of years in Africa, Asia, Europe, and the Middle East, and is used in many commercial preparations for various applications [39,40].

*A. ferox*, also known as bitter aloe or Cape aloe, belongs to the Asphodelaceae family. It is native to South Africa and is widely used in the Western, Southern, and Eastern provinces [41], where it is largely used to treat various diseases, including sexually transmitted infections (STIs) such as gonorrhoea and syphilis [42]. It is a single-stemmed tree that grows to 1.52–1.83 m tall. It has rosettes of canvas-green leaves up to 2½ feet wide and sometimes irregular, sparse tufts of spines [42]. The Food and Drug Administration (FDA) has approved *A. ferox* as a food additive and as a natural flavouring. It is increasingly used in products such as health drinks, beverages, and foods because of its beneficial biological activities [41]. Bitter aloe is used as a laxative in Africa and Europe and is recognised for its bitter tonic, anti-inflammatory, anticancer, antimicrobial, and antioxidant properties [43,44]. Currently, South Africa is the world’s largest producer of wild-harvested aloe bitters, and rural people can benefit economically from the commercial growth of the aloe-tapping business [43].

While many *Aloe* species have demonstrated medicinal and commercial potential, *A. ferox* possesses recognised medicinal and commercial value and is particularly unique in South Africa for its high phytochemical diversity, extensive traditional use, and economic importance. Research has focused mainly on its applications in pharmaceuticals, cosmetics, and nutraceutical products, while crop protection has received comparatively less attention. This imbalance creates an opportunity, as many secondary metabolites important for their medicinal properties are also linked to defensive properties against herbivores and pathogens in plants. As a result, the known ethnobotanical uses and market availability of *A. ferox* provide a valid scientific basis for exploring its potential beyond traditional medicine, particularly for the sustainable use of a botanical insecticide to control stored-grain pests.

## 6. Phytochemistry of Aloe

The phytochemicals and nutrients found in aloe plants include amino acids, indoles, proteins, dicarboxylic acids, chromones, minerals, organic compounds, polysaccharides, flavonoids, anthrones, ketones, enzymes, carbohydrates, alkaloids, pyrones, vitamins, pyrimidines, sterols, phenolic compounds, tannins, glucomannans, alkanes, aldehydes, flavonoids, anthraquinones, alkaloids, saponins, fatty acids, and sterols [45,46]. These compounds support *Aloe’s* important pharmacological properties and enhance its commercial value. For example, aloe plants have been shown to have pharmacological properties, including antibacterial, anti-inflammatory, antiviral, immunomodulatory, antidiabetic, anti-ageing, skin-protective, antioxidant, wound-healing, antifungal, moisturising, antiproliferative, and anticancer properties [45,46].

Compounds such as aloin and aloe-emodin have been shown to possess anti-inflammatory, antiparasitic, antiviral, neuroprotective, laxative, antibacterial, hepatoprotective, and anticancer properties [45,47]. The antioxidant, antibacterial, cytotoxic, and anti-inflammatory activities of aloin against various tumour cell lines have recently attracted pharmacologists’ interest [48]. Aloe-emodin is currently being extensively studied for its exceptional antineoplastic activity against various tumour cell types, including gastric, colon, lung, melanoma, hepatic, and breast cancer cells [47].

The main components of *Aloe* are aloin, aloe-emodin, and aloeresin [49]. *Aloe* roots and leaves contain various phytochemicals, including anthraquinones, alkaloids, anthrones, furans, naphthalenes, steroids, anthraquinones, and pre-anthraquinones [39,48]. Aloin, a major phytochemical constituent of aloe, has the molecular formula of C_21_H_22_O_9_, and the molecular weight is 418.3940 g/mol [48]. Aloe pulp, or parenchyma tissue, contains lipids, flavonoids, vitamins, anthraquinones, inorganic compounds, amino acids, chromones, small organic compounds, anthrones, enzymes, proteins, coumarins, and various carbohydrates [39,50].

**Figure 1:**
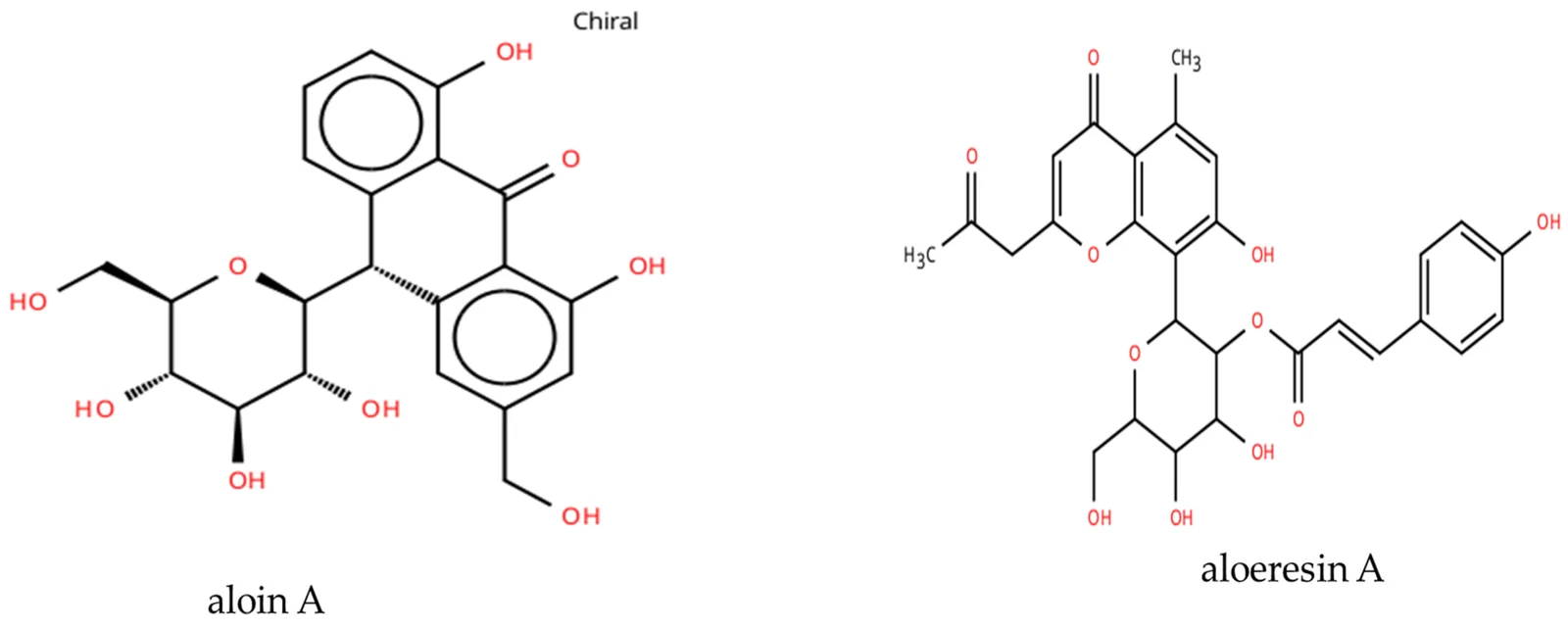
Chemical structure of aloin A and aloeresin A.

*A. ferox* leaf exudate has yielded several bitter compounds. The chromones aloeresin A (Figure 1) and aloesin, as well as the hydroxyanthracene derivative (anthrone derivative) aloin, are the most significant among them [44]. The exudate contains the two diastereoisomers of aloin (A and B) Figure 1, which are among the biologically active components of *Aloe* extracts: aloin A (10S)-10-glucopyranosyl-1,8-dihydroxy-3 (hydroxymethyl)-9(10H)-anthracenone ALNA) and aloin B (10R)-10-glucopyranosyl-1,8-dihydroxy-3-(hydroxymethyl) 9(10H)-anthracenone, ALNB) [44,49,51]. *A. ferox* also contains the anthraquinones aloe-emodin, emodin, rhein, and physcion, which are hydroxyanthracene derivatives. Sugars, amino acids, and organic acids are other components [44].

The phytochemical profile of *A. ferox* also indicates that its biological activity is not due to a single compound but rather the result of the synergistic effects of multiple secondary metabolites. Each compound in anthraquinones, chromones, flavonoids, phenolic compounds, tannins, and saponins has been linked to various pharmacological and biological activities, indicating that the observed bioactivity of anthraquinones is likely the result of interactions among these compounds rather than a single metabolite exerting its activity. Although most attention has focused on aloin and aloe-emodin because of their abundance and broad biological activities, recent studies show that less-studied compounds such as chromones and phenolics may play a significant role in plant defence mechanisms. Moreover, phytochemical profiles vary significantly among *Aloe* species and even within different populations of *A. ferox* because of varying environmental conditions, harvesting seasons, plant ages, and extraction methods. Therefore, understanding phytochemical variability in *A. ferox* is useful for developing reproducible botanical insecticides, since phytochemical variation can affect efficacy and the consistency of pest control.

The biological activities reported do not necessarily indicate insecticidal activity. For several constituents, evidence is limited to their occurrence in *A. ferox* or activity in non-insect biological systems; therefore, their efficacy in *S. zeamais* remains to be investigated rather than proven (See Table 1).

Experimental research findings of different *Aloe* species in relation to pesticidal activities against insects and other arthropods are summarised in Table 2. Most of this evidence comes from *A. vera* and from organisms other than S. zeamais, and should therefore be viewed as comparative rather than conclusive for *A. ferox*. However, the spectrum of insecticidal, repellent, acaricidal, larvicidal, and antifeedant responses provides a rationale for directly assessing the activity of *A. ferox* against *S. zeamais* under laboratory and realistic storage conditions.

The mechanisms summarised in Table 3 here represent different levels of evidence. Unless otherwise stated, they have not been experimentally proven to be valid mechanisms of action for *A. ferox* against *S. zeamais* and thus are regarded as proposed/ indirect mechanisms of action.

## 7. Potential of *A. ferox* Against *S. zeamais*

Experimental evidence for the insecticidal activity of *A. ferox* against *S. zeamais* is scarce. Studies on *Aloes* have demonstrated larvicidal, growth-regulating, repellent, and feeding deterrent effects against several arthropod pests [55,56]. The activity reported in the present studies, however, should be regarded only as indirect evidence when discussing the activity of A. ferox against S. zeamais, especially for other Aloe species and target pests.

*A. ferox* is characterised by the presence of various classes of secondary metabolites such as anthraquinones, steroids, flavonoids, anthrones, pyrones, phenolic compounds, tannins, naphthalenes, alkaloids, glycoproteins, and chromones [46,83,84]. Many of these compound classes have been reported to exhibit insecticidal, repellent, antifeedant, or developmental activities in other insect systems. For instance, flavonoids have been reported to exhibit repellency, feeding deterrence, and developmental disruption in *S. zeamais* [80], while phenolic compounds, alkaloids, tannins, and saponins have been reported to possess various insect toxicity and defence responses [85,86,87]. However, the studies here do not prove that *A. ferox* extracts have the same effects on *S. zeamais*.

Overall, the available evidence provides a rationale for direct experimental testing of *A. ferox* against *S. zeamais* but does not establish efficacy for stored-grain protection.

## 8. Structure–Activity Relationship of Major *A. ferox* Constituents

*A. ferox* is known to be biologically active with its chemically diverse group of specialised metabolites, such as anthrones, anthraquinones, chromones, and phenols. Of these, aloin A and aloin B are regarded as being the major constituents of the exudate of the leaves. The exudate of *A. ferox* contains about 70–97% of aloeresin A, aloesin, and aloin A/B on a dry weight basis, according to a geographical study, and the four compounds have been consistently found at high concentrations in 101 samples by using UHPLC–MS (ultra-high-performance liquid chromatography) [88,89].

### 8.1. Anthrones and Anthraquinones

**Figure 2:**
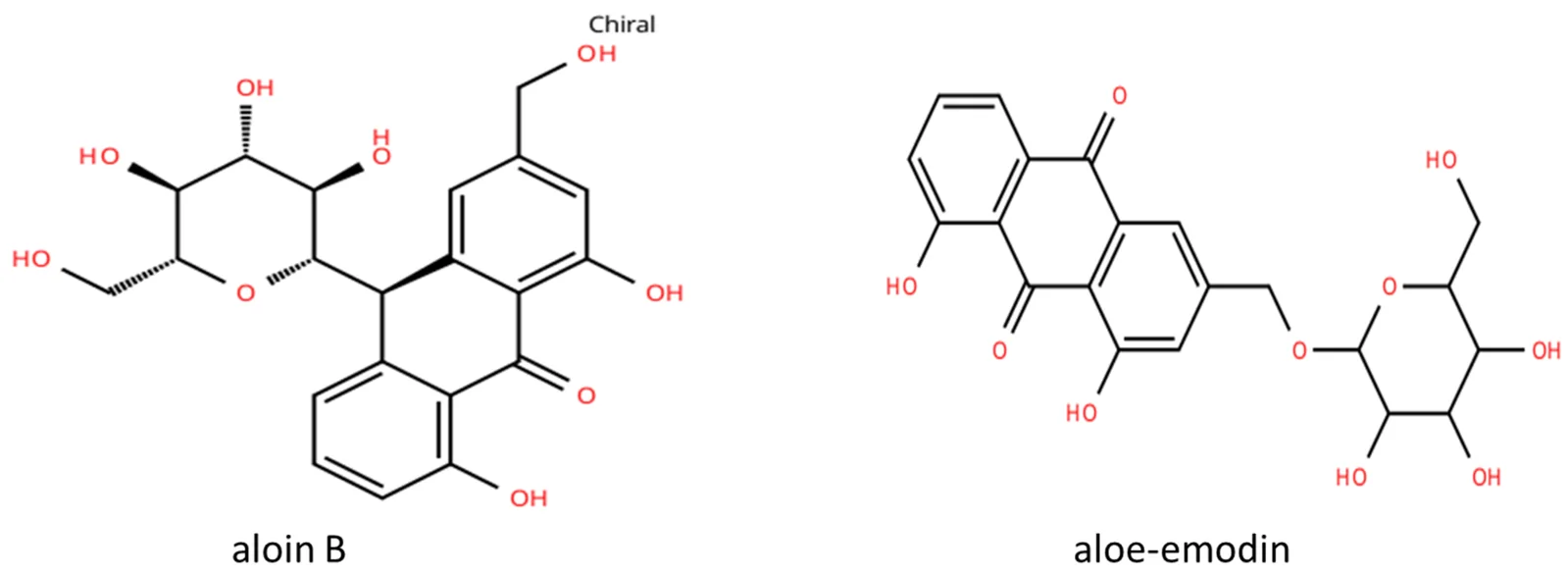
Chemical structures of aloin B and aloe-emodin.

**Figure 3:**
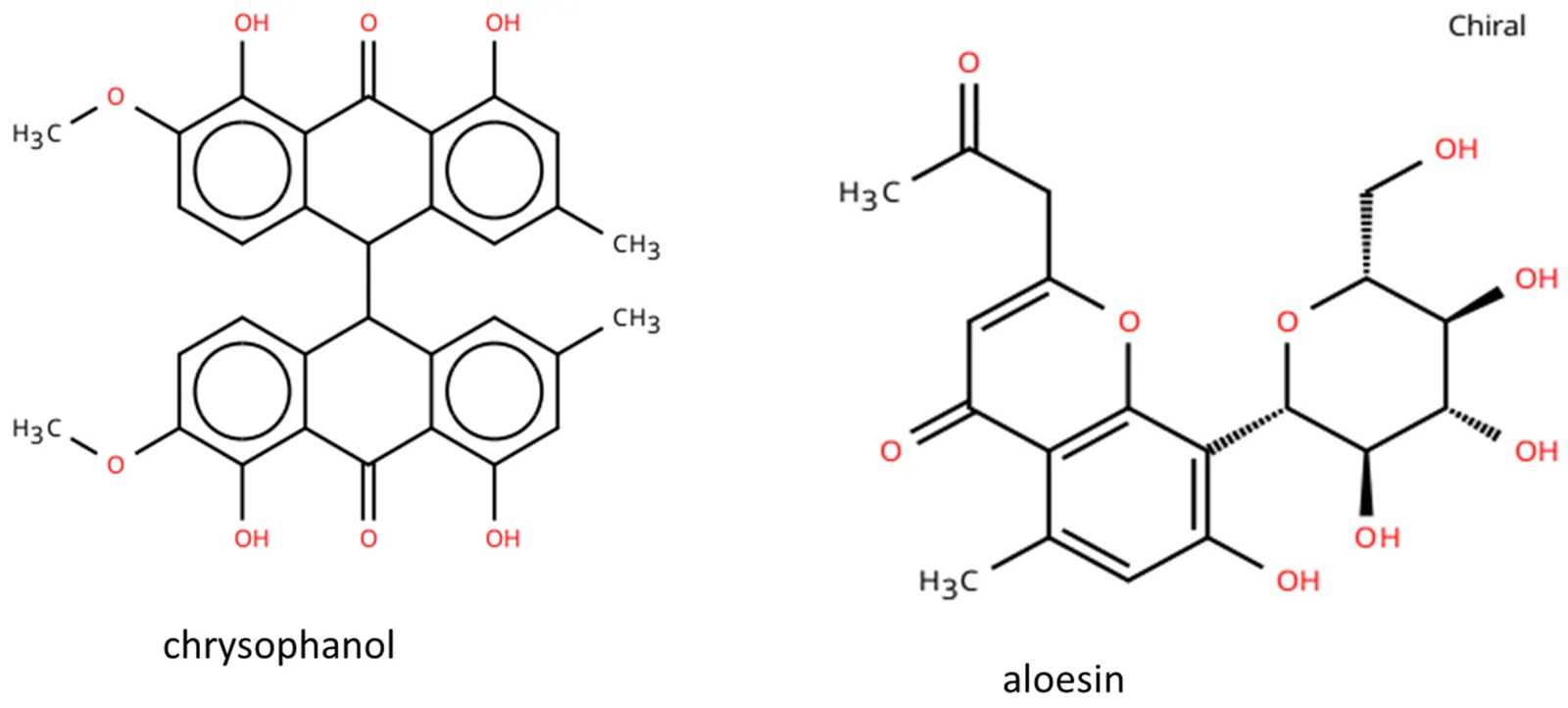
Chemical structures of chrysophanol and aloesin.

Among the most extensively investigated constituents of *A. ferox* are anthrones and anthraquinones. Aloin A and B (Figure 2) are anthrone C-glycosides, while aloe-emodin (Figure 2) and chrysophanol (Figure 3) are anthraquinones. Their structural differences include the oxidation state of the central ring and the types of substituents on the aromatic system. The chemical structure of aloin A is the β-D-glucopyranosyl substituent attached to the anthrone skeleton by a C–C bond; the chemical structure of aloe-emodin and chrysophanol is the anthraquinone carbonyl system. Structural differences affect molecular polarity and other physicochemical properties, and they can be compared in terms of biological activity [43,52].

Kambizi et al. isolated aloe-emodin, chrysophanol, and aloin A from *A. ferox* leaves, providing direct evidence of the biological activity of the isolated anthrones and anthraquinones. Aloe-emodin and aloin A inhibited all six species of bacteria tested, while chrysophanol inhibited three of the tested organisms. In this study, the minimum inhibitory concentrations of aloe-emodin and aloin A ranged from 62.5 to 250 µg/mL, depending on the organism [52]. The results show that the biological profile of structurally related constituents can vary.

These differences may relate to structure, such as the carbonyl group of anthraquinones and hydroxyl substitution, or the presence or absence of a glycosyl group. Note: The observations do not demonstrate an insecticidal SAR. The reported antibacterial activities of aloe-emodin, chrysophanol, and aloin A cannot be directly extrapolated to *S. zeamais*. Instead, they select structurally defined components that can be studied directly against the insect.

### 8.2. Chromones

The other class of abundant constituents of *A. ferox* are called chromones. The main compounds are aloesin (Figure 3) and aloeresin A, as well as several structurally related compounds. Most *A. ferox* chromones share an 8-C-glucosyl-7-hydroxy-5-methyl-2-propyl-4-chromone framework, with structural variation arising from side-chain oxidation, methylation, and glucose esterification [43].

These compounds are quantitatively important in *A. ferox*. UHPLC–MS analyses of 101 exudates of *A. ferox* revealed high levels of aloesin (111.8–561.8 µg/mg) and aloeresin A (129.0–371.6 µg/mg). Aloin A and aloin B were found in small quantities [88]. Biological activity is also shown at the compound level for aloesin. Mikayoulou et al. extracted aloesin and aloeresin A from *A. ferox* and tested them for anti-tyrosinase activity. Aloesin inhibited the enzyme with moderate activity (IC50 = 31.5 µM), while aloeresin A was identified as a substrate of mushroom tyrosinase, not just an inhibitor [90].

The results suggest that the structure of related chromones may affect their interaction with biological targets. However, the anti-tyrosinase activity should not be considered as insecticidal activity. The toxicity, repellency, or fumigant activity of the chromone scaffold or specific substituents to *S. zeamais* still has to be determined experimentally.

### 8.3. Flavonoids and Phenolic Compounds

*A. ferox* also has a variety of structural diversity with phenolic compounds and flavonoids. Compounds reported in Aloe material include catechin, epicatechin, chlorogenic acid, sinapic acid, and aloe-emodin-8-O-β-D-glucopyranoside. These were identified and quantified in a comparative phytochemical study of seven *Aloe* cultivars, which identified aloin, aloe-emodin-8-O-β-D-glucopyranoside, catechin, epicatechin, sinapic acid, and chlorogenic acid [53]. They are characterised by phenolic hydroxyl groups and, in some cases, glycosyl or ester substituents that affect polarity and molecular interactions.

Phenolic compounds can have multiple hydroxyl groups, which can affect the physicochemical and biological properties, along with other substitutions such as glycosylation. However, the available literature on *A. ferox* does not provide enough evidence to identify these structures specifically as responsible for insecticidal activity against *S. zeamais*. Thus, these constituents can be considered potential bioactive constituents but are not established as such.

### 8.4. Volatile Constituents

The volatile fraction of *A. ferox* is chemically distinct and could be significant for managing stored-product insects, as it may act via vapour-mediated and/or behavioural effects. Twenty-one compounds have been identified in *A. ferox* leaf volatile oil, which constitutes over 99.99% of the total volatile matter. The major constituents included 3,6-octatriene (23.86%), 3-cyclohexene-1-methanol (7.31%), bornylene (5.24%), 1,3-cyclopentadiene (4.07%), and 5-methyl-3-heptanol (3.92%) [54].

These constituents are more volatile and have lower molecular masses than the less volatile anthrones, anthraquinones, and chromones. These properties make the volatile fraction worth evaluating for fumigating or repelling stored-product insects. The *A. ferox* volatile oil study, however, was mainly a chemical characterisation study, and not proof of toxicity or repellence for *S. zeamais*. As a result, its role in *A. ferox* insecticidal activity remains an important research gap.

### 8.5. Overall Structure–Activity Implications

Overall, the A. ferox chemical profile suggests that certain structural elements may play significant roles in biological activity, such as oxygenated aromatic systems, hydroxyl and carbonyl groups, C-glycosylation, esterification, and molecular volatility. Experimental models have shown biological activity for isolated *A. ferox* constituents, with aloe-emodin, chrysophanol, aloin A, and aloesin showing activity in antibacterial or enzyme-inhibition models, primarily against *S. zeamais* [43,46]. Results of several *A. ferox* isolated constituents are biologically active in non-insect experimental systems, but these cannot be directly extrapolated to *S. zeamais*.

## 9. Safety and Environmental Considerations of *A. ferox* as a Botanical Insecticide

*A. ferox* extract is known to have a different phytochemical composition depending on the method of extraction, plant part, solvent, growth stage, and plant source. Anthraquinones, flavonoids, tannins, sterols, alkaloids, and volatile oils have been reported as constituents. Toxicological effects have also been observed to differ across preparations of *A. ferox*, such as aqueous leaf extract, leaf resin, and other extracts [46]. It must be noted that the potential use of *A. ferox* as a botanical insecticide does not mean that it is an inherently safe pesticide. Thus, the safety of an *A. ferox*-based insecticidal preparation should be assessed not based on the use of the plant for medicinal purposes or traditional use, but on the extraction method, chemical composition, dosage, mode of application, and formulation of the product.

Experimental evidence on the oral toxicity of *A. ferox* is limited and relates to particular preparations. Celestino et al. evaluated the acute oral toxicity of *A. ferox* resin in Wistar rats after a single dose of 5.0 g/kg, and found that its hydroxyanthracene derivative (as aloin) was 33.5%. No mortality or other signs of acute toxicity were seen during the 14-day observation period, except for moderate diarrhoea and reduced motor activity in the first hour. As a result, it was not possible to calculate the LD_50_ and was therefore regarded as likely to be above 5.0 g/kg. The authors found the resin to be of low acute oral toxicity but noted the need for additional studies on chronic and reproductive toxicity, mutagenicity, and carcinogenicity. Therefore, this study does not demonstrate the safety of a specific *A. ferox* resin product or repeated dietary exposure to *A. ferox*-treated grain [91].

Hydroxyanthracene derivatives are special because they are represented by an important part of the preparations of various *Aloe* species, such as *A. ferox* and *A. vera*. EFSA remarked that hydroxyanthracene derivatives, such as emodin, aloe-emodin, and danthron (which have similar structures), exhibited genotoxic potential in vitro. In addition, aloe-emodin has been demonstrated to be genotoxic in vivo on colon cells, and the whole-leaf *A. vera* extract exhibited carcinogenic activity in experimental animal studies. On this basis, EFSA concluded that hydroxyanthracene derivatives should be considered genotoxic and carcinogenic unless specific data indicate otherwise (e.g., for rhein), and identified a safety concern for extracts containing hydroxyanthracene derivatives, but noted remaining uncertainties. Characterisation and control of the hydroxyanthracene constituents present in botanical formulations used in food commodities is of great importance, as these compounds were identified in these products [92].

The effects on grain germination also need to be explored, especially if maize is stored and later used for seeding. Mbombo and Ngobese conducted an experiment to determine the allelopathic effect of aqueous extracts of *A. ferox* and other plant species on germination of various plants, including *Zea mays*. The study found that all extracts inhibited maize germination compared with the untreated control. Importantly, maize treated with 100% crude *A. ferox* extract achieved 85% germination by day 21, with stronger inhibition noted for some of the other plant extracts (e.g., Anthocleista grandiflora with 54% germination) [93]. This experiment directly applied seeds to aqueous extracts and was not a stored-grain insecticide safety study. Hence, the results should not be taken as an indication of any damage to stored maize caused by treatment with *A. ferox*. They do, however, agree that germination and seed viability should be tested when selecting appropriate concentrations and application methods for stored-grain protection.

The safety of any intended *A. ferox*-based grain protectant should be evaluated for the specific formulation, concentration, method of application, and exposure scenario, including evaluations of residues, dietary and grain-quality, and non-target assessments.

## 10. Research Gaps and Future Directions

Although interest in botanical insecticides is growing, critical knowledge gaps hinder the use of *A. ferox* as a viable grain protectant. First, experimental results comparing *A. ferox* extracts with S. zeamais are scarce and partly based on data from other Aloe species and insect pests. Second, the phytochemical composition of *A. ferox* depends on geographical origin, environmental conditions, plant age, harvest season, and extraction method, and only a handful of studies examine how these sources of variation affect insecticidal activity or product consistency. Third, extraction methods are not well standardised and cannot be easily compared across published studies.

Additional research is required to determine the optimal formulation, application rate, and application method for stored-grain protection under realistic storage conditions. Most published work has been performed under controlled laboratory conditions, while information on semi-field and commercial storage systems remains limited. In addition, little data are available on the long-term insecticidal activity in grain during storage, compatibility with integrated pest management programmes, effects on grain germination and quality, and potential effects on non-target organisms.

Mechanisms of interaction among the major phytochemicals of *A. ferox* that underlie the insecticidal effects should also be explored, as these compounds could act synergistically to significantly contribute to insecticidal activity. Last but not least, economic feasibility, regulatory aspects, product standardisation, and market scalability must be considered alongside biological effectiveness to move *A. ferox* from laboratory research to practical implementation in sustainable stored-grain protection. The potential of nanoformulations, microencapsulation, emulsifiable concentrates, and controlled-release formulations of *A. ferox* remains largely unstudied.

## 11. Conclusions

The biological activities of the secondary metabolites of *A. ferox* make it an interesting candidate for a botanical insecticide. However, direct experiments proving its effectiveness against *S. zeamais* remain insufficient. Comparative and mechanistic evidence from other *Aloe* species, other arthropod pests, and individual phytochemicals is useful but does not establish *A. ferox* efficacy against this stored-grain pest.

Therefore, it is recommended that *A. ferox* be investigated further before being used as a protectant for stored grains. Prioritise direct efficacy and dose–response studies on *S. zeamais*, phytochemical and formulation standardisation, residue and safety evaluations, and grain-quality evaluation. Once the treatment is deemed effective in the laboratory, it must be tested under realistic storage conditions before practical or commercial application.

## Figures and Tables

**Table 1:** Major phytochemical constituents of Aloe ferox and their reported biological activities and relevance to insecticidal research.

Phytochemical Constituent/Class	Chemical Class	Polarity/Nature	Reported Biological Activity	Relevance to Insecticidal Activity	References
Aloin A	Anthrone C-glycoside	Polar	Demonstrated antibacterial activity against several Gram-positive and Gram-negative bacteria, including *Bacillus subtilis*, *B. cereus*, *Staphylococcus aureus*, *S. epidermidis*, *Escherichia coli*, and *Shigella sonnei*.	Its demonstrated biological activity makes it a candidate for further insecticidal investigation; however, direct activity against *S. zeamais* has not been established.	[52]
Aloin B	Anthrone C-glycoside	Polar	Reported as one of the major constituents of *A. ferox* leaf exudate.	As a major constituent, it warrants investigation in insecticidal assays, although direct insecticidal activity against *S. zeamais* remains unconfirmed.	[43]
Aloe-emodin	Anthraquinone	Intermediate/moderate	Isolated from *A. ferox* and showed antibacterial activity against all tested organisms, with MIC values ranging from 62.5 to 250 µg/mL.	Its biological activity and anthraquinone structure support further investigation against storage insects, but direct *S. zeamais* activity has not been demonstrated.	[52]
Chrysophanol	Anthraquinone	Relatively non-polar	Isolated from *A. ferox* and showed antibacterial activity against *B. subtilis*, *S. epidermidis*, and *E. coli*.	Provides evidence that individual anthraquinones from *A. ferox* possess biological activity; insecticidal activity requires direct validation.	[52]
Aloesin	Chromone	Polar/intermediate	Reported as a characteristic constituent of *A. ferox*; chromone constituents of *A. ferox* have been associated with several biological activities.	Its occurrence as a major specialised metabolite makes it relevant for SAR (structure–activity relationship) studies; direct activity against *S. zeamais* remains inadequately investigated.	[43]
Aloeresin A	Chromone derivative	Polar	One of the major constituents of *A. ferox* leaf exudate and a characteristic chromone constituent of *A. ferox*.	Its abundance and biological activity make it an important candidate for compound-level insecticidal testing.	[43]
Aloeresin E	Chromone derivative	Polar/intermediate	Identified in *A. ferox* leaf extracts by HPLC-MS (high-performance liquid chromatography).	Expands the range of chromone constituents relevant to SAR analysis; specific insecticidal activity remains unestablished.	[46]
Isoaloeresin D	Chromone derivative	Polar/intermediate	Identified in *A. ferox* leaf extracts by HPLC-MS.	Potential contributor to extract bioactivity; direct insecticidal activity has not been demonstrated.	[46]
2′-O-Feruloylaloesin	Chromone/phenolic derivative	Polar	Identified among phenolic/chromone constituents of *A. ferox*.	Its structural features make it relevant for SAR comparisons, but insecticidal activity remains unknown.	[46]
Aloe-emodin-8-O-β-D-glucopyranoside	Anthraquinone glycoside	Polar	Identified and quantified among phenolic constituents of *Aloe* cultivars, including *A. ferox*.	Glycosylation may modify polarity, absorption, and biological activity; direct insecticidal activity requires investigation.	[53]
Catechin	Flavan-3-ol	Polar	Identified and quantified in *A. ferox* leaves and contributes to the phenolic profile of the plant.	Phenolic compounds may contribute to extract bioactivity, but *A. ferox*-derived catechin has not been directly demonstrated to control *S. zeamais*.	[53]
Epicatechin	Flavan-3-ol	Polar	Identified and quantified in *A. ferox* leaves.	Relevant to the phenolic/SAR profile; direct insecticidal activity against *S. zeamais* remains unconfirmed.	[53]
Chlorogenic acid	Phenolic acid	Polar	Identified and quantified in *A. ferox* leaves.	May contribute to overall biological activity of extracts; specific insecticidal effects require experimental confirmation.	[53]
Sinapic acid	Phenolic acid	Polar	Identified in *A. ferox* leaves as part of its phenolic profile.	Provides an additional phenolic constituent for SAR consideration; direct insecticidal activity remains unconfirmed.	[53]
Lucenin II	Flavonoid C-glycoside	Polar	Identified among the flavonoid constituents of *A. ferox* leaves by HPLC-MS.	Relevant to flavonoid-based SAR assessment; direct insecticidal activity has not been established.	[46]
Vicenin II	Flavonoid C-glycoside	Polar	Identified among the flavonoids present in *A. ferox* leaves.	Provides a structurally distinct flavonoid for future bioactivity testing; direct activity against *S. zeamais* is unknown.	[46]
Orientin	Flavonoid C-glycoside	Polar	Identified in *A. ferox* leaves by chromatographic profiling.	Potential contributor to biological activity, but insecticidal activity specifically associated with *A. ferox* has not been demonstrated.	[46]
Caffeoylquinic acid derivatives	Phenolic acids	Polar	Caffeoylquinic acid derivatives have been identified among the phenolic constituents of *A. ferox*.	Their phenolic functionality makes them relevant to SAR discussions, although compound-specific insecticidal evidence is lacking.	[46]
Tannins	Polyphenolic phytochemical class	Polar	Detected in *A. ferox* extracts through phytochemical screening; condensed tannins and gallotannins have also been reported.	Tannins may contribute to biological activity through interactions with proteins and digestive processes, but specific insecticidal effects of *A. ferox* tannins require further characterisation.	[46]
Saponins	Glycosidic phytochemical class	Polar and non-polar (amphiphilic)	Detected in *A. ferox* extracts during qualitative phytochemical screening.	Saponins can have membrane-disrupting and antifeedant effects in some systems; however, direct activity of isolated *A. ferox* saponins against *S. zeamais* remains insufficiently documented.	[46]
Alkaloids	Nitrogen-containing phytochemical class	Polar/intermediate	Alkaloids have been detected in *A. ferox* through qualitative phytochemical screening.	Alkaloids are recognised for diverse biological activities, but individual alkaloids from *A. ferox* and their insecticidal effects require further chemical characterisation.	[46]
3,6-Octatriene	Volatile hydrocarbon	Volatile/non-polar	Identified as the major volatile constituent of *A. ferox* leaf volatile oil (23.86%).	Its volatility makes it relevant to possible fumigant or repellent effects, but activity against *S. zeamais* has not been directly demonstrated.	[54]
3-Cyclohexene-1-methanol	Volatile alcohol	Volatile/intermediate	Identified among the major volatile constituents of *A. ferox* leaf oil (7.31%).	Potentially relevant to volatile-mediated biological activity; direct insecticidal evidence is lacking.	[54]
Bornylene	Monoterpene hydrocarbon	Volatile/non-polar	Identified in *A. ferox* volatile oil (5.24%).	Its volatility makes it relevant to potential fumigant/repellent activity, but its activity against *S. zeamais* requires direct testing.	[54]
5-Methyl-3-heptanol	Volatile alcohol	Volatile/intermediate	Identified among the volatile constituents of *A. ferox* leaf oil (3.92%).	May contribute to the oil’s volatile-mediated activity; compound-specific insecticidal activity remains unconfirmed.	[54]
1, 3-cyclopentadiene	Volatile hydrocarbon	Volatile/non-polar	Identified among the volatile constituents of *A. ferox* leaf oil (4.07%).	May contribute to the oil’s volatile-mediated activity; compound-specific insecticidal activity remains unconfirmed.	[54]

**Table 2:** Experimental evidence of pesticidal activities of *Aloe* species against insect and arthropod pests.

*Aloe* Species/Plant Part	Target Pest (Insect Group)/Order	Solvent/Reported Bioactivity	Dose/Concentration/Exposure Time	Effect/LC_50_/LD_50_	Experimental Condition/Evidence Level	Major Phytochemicals	Major Findings	References
*A. vera*/leaves	*Aedes aegypti* (mosquito/vector)/Diptera	Petroleum ether/larvicidal activity	80, 160, 240, 320, and 400 ppm.	162.74, 201.43, 253.30, and 300.05 ppm	Laboratory/related *Aloe*	Not reported	The study clearly stated that *A. vera* could serve as a potential larvicidal agent.	[55]
*A. vera*/leaves	*Hyalomma dromedarii* (tick/ectoparasite)/*Ixodida*	Ethanol/acaricidal and growth regulator	25%/60 s.	88.5% mortality rate (median lethal conc. 7.8%; median lethal time 6.09%)	Laboratory/related *Aloe*	Polyphenols and flavonoids	*A. vera* is a promising acaricide.	[56]
*Aloe adigratana*, *A. vera*, *Aloe berger*, and *Aloe laterita*/leaves	Termites (termite)/Blattodea	Aqueous, powder and mulch/termiticidal	Aqueous solution (2 L/9 m^2^), powder (2 kg/9 m^2^).	Up to 93.98% damaged plants per plot	Field experiment/related *Aloe*	Not reported	The best results were consistently obtained when *Aloe adigratana* was utilised as mulch.	[57]
*A. vera*/leaves	*Callosobruchus maculatus* (stored-product pest)/Coleoptera	Powder/insecticidal (contact toxicity)	0 g (control), 0.5 g, 1.0 g, 1.5 g, 2.0 g, and 2.5 g/20 g of cowpea seeds at 48 h and 30 days post-treatment.	Contact toxicity. 10.33% maximum mortality at 2.0 g/20 g led to a 3.33% reduction in seed damage. LC_50_/LD_50_ (not reported)	Laboratory/related *Aloe*	Not reported	No mortality caused by *A. vera* leaf powder was found in *C. maculatus* after 48 h exposure. In contrast, it was concentration-dependent and reduced seed damage after 30 days of exposure. Treatment at 2.0 g resulted in the highest mortality (10.33%) and the greatest reduction in seed damage (3.33%), indicating low insecticidal efficacy.	[58]
*A. vera*/leaves	*Callosobruchus maculatus* (stored-product pest)/Coleoptera	Aqueous/insecticidal (biological and lethal effects)	(Concentration: 0, 0.5, 1, 1.5, 2, and 3%). Mortality evaluated at 24, 48, and 72 h.	Minimum of 13.89% mortality at 0.5% conc. after 48 h; maximum of 52.78% mortality at 3.0% conc. after 72 h. LC_50_/LD_50_ (not directly reported)	Laboratory/related *Aloe*	Not reported	After 72 h of treatment, there is a maximum rate of mortality (52.78%) in cowpea seeds treated with 3% aloe aqueous extract.	[59]
*A. vera*/leaf ash, leaf powder, root ash, and root powder	*Tribolium castaneum* (stored-product pest)/Coleoptera	Ash and powder/insecticidal and insect productivity	1 g, 2 g, and 5 g. Mortality (within 7 days). Percentage weight loss (within 30 days).	Mortality % was based on concentration and plant part used	Laboratory/related *Aloe*	Not reported	The findings demonstrate that *T. castaneum* was adversely affected by both low and high dosages, and no insect productivity rate was observed because the insects died before they could lay eggs.	[60]
*Aloe ngongensis*, *Aloe turkanensis*, and *Aloe fibrosa*/leaves	*Aedes aegypti* (mosquito/vector)/Diptera	Hexane, chloroform, ethyl acetate, acetone, and methanol/larvicidal	0.05 to 2.0 mg/mL at 24 h.	Highest activity was obtained from A. fibrosa hexane (0.05 [0.04–0.06])	Laboratory/related *Aloe*	Not reported	Depending on the extraction solvent utilised, the actions appeared to be unique to various Aloe species. These results suggest the presence of mosquitocidal compounds in extracts from specific Aloe species, which may be used to develop novel insecticides.	[61]
*A. vera*/gel, root, and leaf peel	*Sitophilus oryzae* (stored-product pest)/Coleoptera	Methanol/repellent and contact toxicity	0.0024 mg/cm^2^ for aloin A and 0.060 mg/cm^2^ for plant extracts in toxicity test; 0.02% *w*/*v* and 5% *w*/*v* in repellency tests). Repellency (1 h, 5 h, and 24 h exposure. Contact toxicity (14 h. days after treatment).	Standard aloin A (58.0%) mortality	Laboratory/related *Aloe*	Aloin A	Geographical location, plant sections, main metabolite concentration, and exposure duration all affect the percentage of repellency and toxicity of *A. vera*. These plant extracts could help develop an efficient biorepellent for S. oryzae in stored maize.	[62]
*A. vera*/leaves	*Bactrocera cucurbitae* (fruit pest) and *Myzus persicae* (sap-sucking crop pest)/Diptera, Hemiptera	Aqueous/insecticidal	40, 60, and 80% at 24 h, 48 h, and 72 h post-exposure.	100% mortality at 80% and 60% after 48 h and 72 h, respectively, for the two insects	Laboratory/related *Aloe*	Not reported	The findings show that *A. vera* extracts, compared with untreated crops, may help manage insect pests.	[63]
*A. vera*/leaves	*Raphidipulpa foveicollis* (leaf-feeding or defoliating insect)/Coleoptera	Aqueous/pesticidal	0, 25, 50, 75, and 100%. 12 h., 24 h., and 48 h.	The highest mortality was 71.33% at 100% concentration	Laboratory/related *Aloe*	Not reported	*A. vera* may be beneficial in managing insect pests.	[64]
*A. vera*/leaves	*Tetranychus urticae* (plant-feeding mite) and *Cenopalpus pulcher* (plant-feeding mite)/Trombidiformes	Acetone/acaricidal	Concentration not explicitly reported at 72 h.	Acetone extract of both species has a toxicity index of 100%, while the water extract for *C. pulcher* has the lowest toxicity index of 25%/ LC_50_ *T. urticae* (acetone: 105 ppm, ethanol 322 ppm, and water 366 ppm). *C. pulcher* (acetone:80 ppm, ethanol:289 ppm, and water 320 ppm)	Laboratory/related *Aloe*	Not reported	The results showed that *A. vera* has significant potential as a botanical acaricide for controlling *C. pulcher* and *T. urticae*.	[65]
*A. vera*/leaves	*Trogoderma granarium* (stored-product pest)/Coleoptera	Methanol and gum resin powder/repellency and fumigant toxicity	Repellency (0.5, 0.9, 2 and 4 *w*/*v*). Fumigant (0.2, 0.4, 0.6, and 0.8%) at 24, 48, and 72 h.	Repellency mortality at 4% after 24 h was 76.7%, and the lowest mortality ranged from 11 to 24.67% at 4% after 72 h. Fumigant mortality at lower conc. after 24 h was 0% and 100% at 0.8%. After 72 h, mortality ranged from 70% (at 0.2%) to 100% (at 0.8%)	Laboratory/related *Aloe*	Not reported	*A. vera* can be beneficial in developing a natural pesticide.	[66]
*A. vera*/leaves	*Tetranychus cinnabarinus* (plant-feeding mite)/Trombidiformes	Acetone, methanol, absolute ethanol, petroleum ether, and ethyl acetate/acaricidal	Concentrations (2500, 1250, 625, 312.5, and 156.25 ppm) at 24 h, 48 h, and 72 h.	Strongest acaricidal activity was 100% at an LC_50_ of 99 ppm after 72 h, while LC_50_ values for ethyl acetate, water, and ethanol extracts were 113, 340, and 391 ppm, respectively	Laboratory/related *Aloe*	Not reported	According to the findings, *A. vera* has high potential for development as a botanical acaricide for controlling *Tetranychus cinnabarinus*.	[67]
*A. vera*/leaves	*Papilio polytes* (leaf-feeding or defoliating pest)/Lepidoptera	Aqueous/deterrent, antifeedant, and ovicidal	0.5, 0.4, 0.3, 0.2, 0.1, 1.0, 3.0, 5.0, and 10.0% at 72 h.	Deterrent activity 11%, antifeedant 26%, ovicidal 8%, and adult emergence 91%	Laboratory/related *Aloe*	Not reported	*A. vera* has the lowest deterrent, antifeedant, and ovicidal activity, resulting in the highest adult emergence in comparison to other tested plants	[68]
*A. ferox*/leaves	*Anopheles arabiensis* (mosquito or vector)/Diptera	Dichloromethane and ethanol/adulticidal	10 mg/mL at 1 h, and mortality recorded after 24 h.	Dichloromethane extract mortality was 98%, and ethanol extract mortality was 86%; EC_50_ was 4.92 mg/mL	Laboratory/direct evidence of *A. ferox*, but not against *S. zeamais*	Not reported	According to the study, *A. ferox* leaf dichloromethane extract may be useful as a pesticide against *An. arabiensis*.	[69]
*A. vera*/leaves	*Spodoptera exigua* (leaf-feeding or defoliating pest)/Lepidoptera	Methanol/toxicity and antifeedant	10% at 7 days post-treatment.	Contact bioassay (13.34%) and feeding bioassay (26.67%). LC_50_/LD_50_ not reported	Laboratory/related *Aloe*	Not reported	The plant was effective, but less effective than the other plants used in this study.	[70]
*A. vera*/leaves	*Bemisia tabaci* (sap-sucking crop pest) and *Aphis gossypii* (sap-sucking crop pest)/Hemiptera	Aqueous/insecticidal	10% (*w*/*v*) sprayed at the rate of 200 L per feddan/post-treatment at intervals of 1 day, 4 days, 7 days, and 14 days.	Seasonal general mean reduction percentage on *B. tabaci* (in 2020, 48.24% and in 2021, 48.89%). *A. gossypii* in 2020 was 56.74% and in 2021, 59.99%	Field experiment/related *Aloe*	Not reported	*A. vera* extract ranked second-to-last among the five tested plant extracts against *B. tabaci* and third against *A. gossypii*.	[71]
*A. vera*/leaves	*Trogoderma granarium* (stored-product pest)/Coleoptera	Ethyl alcohol/insecticidal	0.16%, 0.31%, 0.63%, 1.25%, and 2.5% at 24, 48, and 72 h of post-treatment (mortality) and 1–10 days for residual toxicity effects.	The highest residual toxicity effect was 40% after days 2, 3, and 5, and the highest mortality effect was 63.33% at 1.25% on day 2	Laboratory/related *Aloe*	Not reported	In laboratory settings, *A. vera* showed promise as a safe and efficient phytopesticide.	[72]
*A. vera*/leaves	*Bemisia tabaci* (sap-sucking crop pest)/Hemiptera	Essential oil/contact toxicity	%0.125, 0.25, and 0.5/24, 48, and 72 h.	The mortality effect of *A. vera* oil on eggs and nymphal stages of *B. tabaci*, *O. laevigatus*, *E. formosa*, and *E. eremicus* was 53.33%, 46%, 53%, 46%, and 36%, respectively	Laboratory/related *Aloe*	Not reported	*A. vera* oil was the least effective essential oil tested in this experiment used in this study.	[73]
*A. ferox*/leaves	*Rhipicephalus* (Boophilus) *microplus*, *Amblyomma hebraeum*, and *Rhipicephalus appendiculatus* (tick or ectoparasite)/Ixodida	Distilled water/acaricidal	20 and 40% (*w*/*v*)/0, 1, 2, 3, 4, and 7 days post-application.	*A. ferox* caused 0% mean tick load reduction across all tested tick species	Field experiment/direct evidence of *A. ferox*, but not against *S. zeamais*	Not reported	*A. ferox* was not effective.	[74]
*A. vera*/leaves	*Tetranychus cinnabarinus* (plant-feeding mite) and *Panonychus citri* (plant-feeding mite)/Trombidiformes	Acetone/acaricidal	1.0 mg/mL at 24 h and 48 h, and a range of 5 conc. tested up to 72 h.	Mortality effect on *T. cinnabarinus* was 80.39–92.16%. LC_50_ differs based on the tested insect, plant extract, and the isolate used	Field experiment/related *Aloe*	3-O-alpha-D-mannopyranosyl-D-mannopyranose (OAMM)	Aloe-emodin and OAMM, isolated from an acetone extract of *A. vera*, showed clear acaricidal effects against *P. citri* and *T. cinnabarinus*.	[75]
*A. vera*/leaves	*Onchocerca ochengi* (nematode) and *Caenorhabditis elegans* (nematode)/Spirurida, Rhabditida	Distilled water, methanol, methylene chloride/anthelmintic activity	0–40 µ/mL at 48 and 72 h.	LC_50_ was concentration-dependent	Laboratory/related *Aloe*	Not reported	*A. vera* showed in vitro nematicidal action against *O. ochengi* and *C. elegans*. An alternate anthelmintic for treating onchocerciasis	[76]
*A. vera*/leaves	Anopheles mosquito larvae (mosquito or vector)/Diptera	Gel (no extraction)/larvicidal	0.5, 1.0, 2.0 (mg/mL) at 24 and 48 h.	Mortality was exposure- and concentration-dependent. Highest mortality was at 2 mg/mL after 24 h, while the minimum mortality was at 0.5 mg/mL after 48 h of exposure	Laboratory/related *Aloe*	Not reported	The study showed how effective *A. vera* is at controlling the larvae, making it a cost-effective method of controlling Anopheles mosquitoes’ larvae.	[77]
*A. vera*/leaves	*Culex* sp. (mosquito or vector)/Diptera	Ethanol/larvicidal	20, 40, 60, 80, and 100 ppm/observation time: 180, 360, 540, 1440, and 2880 min.	Lethal concentration median (%) was 68.8 ppm at 1440 min.	Laboratory/related *Aloe*	Not reported	The findings of this study indicate that *A. vera* extract possesses larvicidal properties against *Culex* sp. larvae in instars III and IV.	[78]
*A. vera*/leaves	*Callosobruchus maculatus* (stored-product pest)/Coleoptera	Powder/insecticidal	0.2, 0.5, 1.0, 1.5, and 2.0 (g/mL) at 24, 48, 72, 96, 120, 144, and 168 h.	Mortality was highest at 93.3% at 2.0 g/mL and lowest at 40% at 0.25 g/mL. The LD_50_ and LD_90_ were 0.34 g/mL and 1.72 g/mL, respectively.	Laboratory/related *Aloe*	Not reported	*A. vera* was found to be effective in reducing cowpea pest attacks.	[79]

**Table 3:** Evidence supporting proposed mechanisms of action of phytochemicals relevant to the insecticidal potential of *A. ferox*.

Phytochemical/Class	Source	Target Organism/System	Endpoint Measured	Reported Mechanism/Activity	Level of Evidence for *A. ferox*–*S. zeamais*	References
Aloin A, Aloe-emodin and Chrysophanol	*A. ferox*	*Bacillus cereus*, *B. subtilis*, *Staphylococcus aureus*, *S. epidermidis*, *Escherichia coli*, *Shigella sonnei*	MIC (Minimum Inhibitory Concentration)	Antibacterial activity was demonstrated; the molecular mechanism was not established.	Direct evidence for *A. ferox*, but non-insect system; insecticidal mechanism unknown	[52]
Flavonoids	Various plant species	Various agricultural insects	Mortality, feeding, development, enzyme activity	Depending on the compound, reported effects include feeding deterrence, disruption of digestive enzymes, altered detoxification enzymes, and developmental effects.	Evidence from other insects; mechanism not established for *A. ferox* against *S. zeamais*	[80]
Phenolic compounds	Various plant species	Herbivorous insects	Feeding, growth and digestion	Phenolics and tannins can affect feeding and digestion through interactions with proteins and digestive processes; effects are compound- and insect-dependent.	Evidence from other plant–insect systems; mechanism remains unconfirmed for *A. ferox*–*S. zeamais*	[81]
Steroidal saponins	*Trillium govanianum*	*Plutella xylostella* and *Aphis craccivora*	Mortality, reproductive inhibition and repellency	Steroidal saponins demonstrated insecticidal, repellent, and reproductive inhibitory effects through enzyme activities (glutathione-S-transferase (GST) and carboxylesterase (CES1)	Experimental evidence in other insects; not demonstrated for *A. ferox* against *S. zeamais*	[82]

## Data Availability

No new data were created or analysed in this study. Data sharing is not applicable to this article.

## Abbreviations

The following abbreviations are used in this manuscript:

IPM	Integrated Pest Management
FDA	Food and Drug Administration
EOs	Essential oils
STIs	Sexually Transmitted Infections
MIC	Minimum Inhibitory Concentration
UHPLC-MS	Ultra-High-Performance Liquid Chromatography
HPLC	High-Performance Liquid Chromatography
SAR	Structure-activity Relationship
